# Promoting Employees’ Recovery During Shift Work: Protocol for a Workplace Intervention Study

**DOI:** 10.2196/17368

**Published:** 2020-07-14

**Authors:** Irene MW Niks, Alwin van Drongelen, Elsbeth M de Korte

**Affiliations:** 1 The Netherlands Organization for Applied Scientific Research (TNO) Leiden Netherlands

**Keywords:** shift work, recovery during work, fatigue, intervention study, participatory action research, ecological momentary assessment, mobile phone

## Abstract

**Background:**

Shift work can be demanding owing to disturbances in the biological and social rhythms. This can cause short-term negative effects in employees, such as increased fatigue and reduced alertness. A potential way to counteract these negative effects is to enhance employees’ recovery from work during working hours.

**Objective:**

The aim of this study is to develop and implement an intervention that focuses on promoting “on-job” recovery of shift workers.

**Methods:**

This study is performed in 2 department units with shift workers at a multinational company in the steel industry. For each department, an intervention will be developed and implemented through an iterative process of user-centered design and evaluation. This approach consists of various sessions in which employees and a project group (ie, researchers, line managers, human resource managers, and occupational health experts) provide input on the intervention content and implementation. Intervention effects will be evaluated using pretest and posttest web-based surveys. Digital ecological momentary assessment will be performed to gain insight into the link between the intervention and daily within-person processes. The intervention process and participants’ perception of the interventions will be assessed through a process evaluation. Intervention results will be analyzed by performing mixed model repeated measures analyses and multilevel analyses.

**Results:**

This study is supported by the Netherlands Organization for Applied Scientific Research Work and Health Research Program, which is funded by the Ministry of Economic Affairs and supported by the Dutch Ministry of Social Affairs and Employment, program number 19.204.1-3. This study was approved by the institutional review board on February 7, 2019. From June to August 2019, baseline data were collected, and from November to December 2019, the first follow-up data were collected. The second follow-up data collection and data analysis are planned for the first two quarters of 2020. Dissemination of the results is planned for the last two quarters of 2020.

**Conclusions:**

A strength of this study design is the participatory action approach to enhance the stakeholder commitments, intervention adherence, and compliance. Moreover, since the target group will be participating in the development and implementation of the intervention, the proposed impact will be high. In addition, the short-term as well as the long-term effects will be evaluated. Finally, this study uses a unique combination of quantitative and qualitative evaluation methods. A limitation of this study is that it is impossible to randomly assign participants to an intervention or control group. Furthermore, the follow-up period (6 months) might be too short to establish health-related effects. Lastly, the results of this study might be specific to the department, organization, or sector, which limits the generalizability of the findings. However, as workplace intervention research for shift workers is scarce, this study might serve as a starting point for future research on shift work interventions.

## Introduction

### Background

The expanding 24-hour economy, ongoing globalization, and technological developments have resulted in about 19% of the workforce working during the night and about 21% involved in work schedules with permanent or rotating shifts [1]. In the Netherlands, about 1.2 million employees are involved in shift work, including night shifts, and in sectors such as the heavy industry, the percentage of employees working in shifts is about 25% [2]. Short-term irregular working hours have been reported to lead to fatigue, sleep loss, and an increased accident risk [3,4]. Long-term shift work has been reported to result in severe health issues, including gastrointestinal, reproductive, metabolic, and cardiovascular disorders [5]. Despite these health problems, there are hardly any evidence-based interventions that can mitigate the negative effects of shift work [6]. A potential starting point for interventions to reduce the negative short-term effects of shift work is recovery from work. Recovery from work is defined as the psychophysiological unwinding following effort expenditure at work [7]. When refraining from work demands, activated bodily systems can unwind and return to baseline levels. This process of unwinding has the potential to reduce fatigue, subsequent alertness problems, and accident risks [8]. Research has indeed shown beneficial effects of recovery after work (eg, evenings, weekends), such as decreased burnout, increased performance [9], and lowered levels of blood pressure, heart rates, and epinephrine excretion [10]. However, little research has been performed to investigate the recovery during working hours [11]. Recovery during work may be particularly relevant for shift workers, as it has the potential to directly counteract the increased levels of sleepiness, fatigue, and the associated accident risk. Moreover, it has been shown that good recovery practices can be learned and that differences between individual preferences for recovery practices can be accounted for in recovery interventions [12,13]. In short, recovery during work seems to be a promising direction for tailored interventions that aim to mitigate the negative effects of shift work. In this paper, we will therefore start with a brief discussion of recent literature on recovery research to identify the potentially effective ingredients for recovery interventions. Subsequently, we will describe the development, implementation, and evaluation of a tailored workplace intervention aimed at enhancing recovery during the work of shift work employees.

### Effective Ingredients for Recovery Interventions

#### Psychological Detachment

Studies have shown that a particularly powerful recovery experience is mental disengagement from work [14,15]. This is also referred to as psychological detachment from work or, in everyday terms, it is called as “switching off” [16]. It implies being occupied with things other than work and allows for physiological and psychological restorative processes to occur. In order to achieve this type of recovery, employees should refrain from job-related activities for a certain amount of time. Moreover, they should engage in activities that help them to temporarily take work off their mind. On a more critical note, it remains to be seen to what extent psychological detachment from work is actually possible during the working hours because contact with coworkers and the overall work setting cannot be avoided and therefore, this setting would most likely not result in full detachment [13]. Further, the single time periods for recovery activities are short. Thus, it is important to develop an intervention that allows for recovery activities that encourage psychological detachment during a limited period of time. For instance, some types of recovery activities (eg, mindfulness practices, exposure to a natural environment) may provide some degree of recovery rather quickly [13], while others may require more time and organization (eg, engaging in team sports). Two important types of recovery activities that may foster psychological detachment from work are relaxation and activation [13]. We have discussed both these types in the following section.

#### Recovery Activities: Relaxation and Activation

Relaxation is a positively toned state of low arousal that can benefit people in recuperating after a busy day at work [13]. In terms of relaxation, studies on interventions for shift workers have mainly focused on the recovery effect of napping. Napping allows employees to both physically and mentally detach from work for a short period of time. Richter et al [16] reported that basic conditions such as a pleasant, clean, and undisturbed surrounding for a healthy meal and a silent dark room for taking a nap are essential for the prolonged well-being of shift workers. In addition, the Health Council of the Netherlands [6] indicated that taking a short nap during the night shift may have a positive effect on alertness and may reduce sleepiness and fatigue. However, the Council also concluded that there is not enough knowledge regarding the optimal timing of such a nap. Ruggiero and Redeker [17] found that many individual characteristics such as age, gender, and years of experience can influence the potential positive effects of napping. Naps of 20-40 minutes have been reported to have the most beneficial effects, while the effects of short naps lasting to a maximum of 10 minutes are largely unknown [18]. Despite these potential positive effects, many workers do not apply napping as a recovery activity since they presume that naps take too much time and they fear feeling worse afterwards. Moreover, they feel they are too busy or do not have a comfortable napping space and they feel that the management would not support them in taking a nap during work [18]. We are not aware of articles that describe intervention studies focusing on other types of relaxations or work stress reduction techniques for shift workers.

Detachment from work may also be accomplished by ways other than a reduction in activity (ie, relaxation). There is ample evidence that an increase in social or physical activities can contribute to unwinding from work as well [13,19,20]. For instance, Sianoja et al [21] found that employees experienced less fatigue and high levels of well-being at the end of a working day on which they engaged in recovery activities such as park walks during lunch breaks. Furthermore, social activities during lunch breaks can be conducive to recovery [20] as long as employees have a high sense of autonomy in selecting their social activities during a work break [11]. In other words, a sense of autonomy or control over the break activities during work seems to be one of the basic conditions for recovery to occur [12].

Hardly any study has shown the effect of activation as a means for recovery during shift work. Stimuli such as social interactions, job variations, physical activities such as standing or walking, and exposure to sound and light are thought to increase alertness, but more research is needed to establish the usefulness of such interventions as countermeasures to the negative effects of irregular working hours [22]. Instead, shift work interventions have primarily focused on optimizing the physical capacity of the employees and, thereby, the proposed tolerance of shift workers through off-job training programs [23]. A study has shown that appropriately timed physical exercise may be used to adapt to a certain shift schedule or readapt to a daytime schedule [22]. In one experimental study, physical exercise (2-6 training sessions per week) was found to lead to not only increased physical performance but also increased alertness and increased short-term memory during the night shifts of female shift workers [24]. Although these training sessions may be effective, this intervention may be very difficult to implement during the actual working hours of the employees.

### Study Objectives

Studies have shown that the experience of psychological detachment from work is an effective ingredient for an intervention aimed at enhancing recovery during work [13-15]. This experience may be evoked through either relaxing or activating recovery activities as long as employees have a sense of control with regard to choosing their recovery activities. Nevertheless, the optimal intervention for enhancing recovery during the work of shift workers has not been elucidated in practical settings. Therefore, the main objective of this study is to develop, implement, and evaluate an intervention aimed at improving recovery during work and thereby reduce the negative short-term effects of shift work. Not only the content but also the intervention development and implementation process can affect the outcomes of the respective intervention [25,26]. The evaluation will therefore focus on the effects of the intervention as well as the process of intervention development and implementation. The corresponding research questions of this study are as follows:

1. What is an optimal intervention to enhance recovery during work for shift workers in practice?
2. To what extent is this intervention effective in enhancing recovery during work and in reducing the negative short-term effects of shift work?
3. What are the hindering and facilitating factors for the development and implementation of the intervention?

## Methods

### Ethical Approval and Consent to Participate

This study protocol and materials have been approved by the Netherlands Organization for Applied Scientific Research’s review board, which is an internal ethics committee that assesses the ethical aspects of working with participants in experiments. In addition, both the higher and lower management of the shift work organization provided their consent for the execution of the research plan. Employees will be extensively informed about the study purpose and the protocols and asked to sign an informed consent form before participation. The confidentiality of the research data will be guaranteed and participation will strictly happen on a voluntary basis; thus, participants can withdraw from the study at any moment.

### Quasi-Experimental Field Study

A randomized controlled trial is generally considered as the gold standard in evaluative health-related research, as causal inferences about the therapy under study can be drawn [27]. However, in this project, there are several practical and ethical issues that do not allow for such a design. Most importantly, a precondition set by the participating organization was that their management would select 2 preexisting departmental units for the study and that both units would receive the intervention. However, a control group is needed to distinguish between the change in the outcome over time due to the planned intervention or to evaluate the changes over time due to unmeasured or unknown factors [27]. Therefore, this study was designed as a quasi-experimental field study with a waiting list control group and pretest-posttest design. This means that one part of the experimental group (ie, unit A) will receive the intervention first, while another part of the group (ie, unit B) waits for an additional 3 months before they can make use of the intervention. Thus, the second unit acts as a temporary control group.

Three types of research methods can be distinguished in this study: (1) a longitudinal web-based survey study, (2) an ecological momentary assessment (EMA) study, and (3) a process evaluation. After the baseline measures (T0) of the web-based survey study are recorded, the yet-to-be-developed intervention aimed at recovery during work will be implemented within the experimental group.

**Figure 1:**
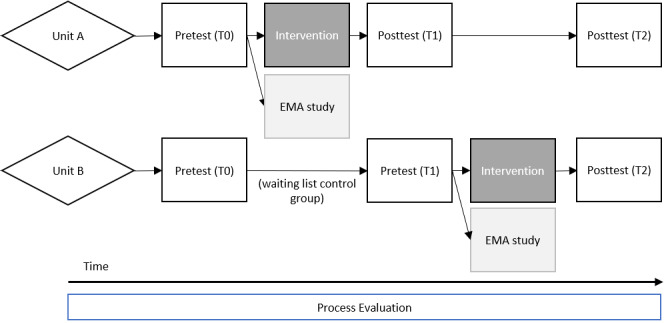
Study design. EMA: ecological momentary assessment; T0: baseline measurements; T1: measurements after 3 months; T2: measurements after 6 months.

Figure 1 presents a flowchart of the study design and the measurement moments. To analyze the effectiveness of the intervention, follow-up measurements will be performed at 3 months (T1) and at 6 months (T2) after the implementation of the intervention. This timeline seems adequate as the primary study outcomes (recovery during work and short-term negative effects of shift work) are expected to change over a relatively short period of time. In addition, an EMA study will be carried out shortly after the implementation of the intervention to provide insight into the short-term within-person intervention effects. Finally, a process evaluation will be performed to determine the factors that may have either enhanced or mitigated the effectiveness of the intervention.

### Setting and Study Population

This study is performed at the logistics department of a multinational steel company. This department consists of 160 employees in rotating shift work who are predominantly men, and they are divided into 2 units. These units do not share physical workspaces and are managed separately, thereby minimizing the chance of potential crossover effects. With respect to the demographics and the workload, the units are highly comparable. The typical job positions in both the units are machine operators and logistics coordinators. Open air working conditions are limited for both the participating units. One of the units will be selected to serve as the waiting list control group. This selection will be made in close consultation with the management, considering factors such as organizational planning, expected availability of time, and resources available for each unit.

### Inclusion Criteria

All questionnaires used during the study will be distributed using an app. Therefore, the only inclusion criteria are that employees have to work for one of the 2 participating units and they need to possess their own personal smartphone to be able to participate in the study.

### Intervention Development and Implementation

The current protocol for intervention development and implementation is based on close alignment between the researchers and human resource (HR)/occupational health representatives of the respective multinational steel company. Before the start of the participatory process (phase 1: needs assessment), the company already signaled issues with respect to fatigue and recovery on the basis of the internal employee surveys. However, as mentioned before, for recovery interventions to be effective, it is essential that employees have a sense of autonomy and control with regard to choosing their recovery activities [11,12]. To provide this sense of control, it seems vital to actively involve the target group in the intervention design and implementation process. In fact, Nielsen et al [28] argued that the effect of occupational health–promoting interventions heavily depends on the participation of the various organizational stakeholders. Therefore, in this study, a bottom-up participatory action research (PAR) approach will be used for intervention development and implementation [29]. By establishing a participatory group and making use of the management’s and workers’ knowledge, skills, and perceptions, a feeling of joint ownership of both problems and solutions is created [30]. Further, involving organizational stakeholders in the process of intervention development and implementation may enhance their general capacity to successfully address workplace issues [29]. The participatory group will be led by researchers with expertise in occupational health and PAR approaches. The effectiveness of PAR approaches has been demonstrated in various intervention studies [31,32].

The PAR approach toward intervention development and implementation consists of various sessions for each participating unit, in which employees and a project group (ie, researchers, line managers, HR managers, occupational health experts) provide input on the intervention content and implementation. The development and implementation process can be divided into 3 phases, which are described below.

#### Phase 1: Needs Assessment

In phase 1, we aim to assess the initial design specifications and the user requirements for the intervention to meet the needs of the target group. The basic needs and requirements of the intervention will be determined in close consultation with the target group. To this end, we will apply a user-centered design approach. First, the researchers will observe the workplace of the participating departmental units and conduct semistructured interviews with the employees and departmental and unit managers to gain insight into the characteristics of the target group, their specific work activities, and their current recovery practices. Second, the employees from the participating units (ie, intervention groups) will be invited to participate in 2 successive so-called user sessions that are led by the researchers. In these sessions, evidence-based and theory-based recovery practices (ie, psychological detachment, relaxation and activating recovery activities) will be presented and discussed. Specifically, employees will be asked for their recovery needs (eg, preferred work break activities) in context to specific work shifts (ie, morning, evening, night). In addition, they will be asked for ideas on how to incorporate new recovery practices into their current work and recovery routines. For reasons of feasibility, the focus will be on their formal work breaks. These ideas will then be rated on desirability by all employees. A voting procedure will be used to reach consensus. Phase 1 will result in an overview of the general intervention requirements.

#### Phase 2: Intervention Development

The aim of phase 2 is the development of an intervention prototype that can be tested in practice. To ensure that the intervention will meet the individual needs of various employees, the prototype should include the introduction and facilitation of a minimum of three recovery practices. These practices may be directed at both the individual and the work environment, depending on the outcomes of phase 1. The development of the intervention will again take place in consultation with the project group and the target group (ie, employees of the departmental units A and B). An intervention development and implementation team will be set up, consisting of various target group representatives (ie, employees, managers, and possibly HR advisors and works council members). We will use an iterative design to transform a paper prototype into a working prototype, considering the general intervention requirements that are identified in phase 1. Phase 2 will result in an intervention prototype and an accompanying implementation plan.

#### Phase 3: Intervention Implementation

In phase 3, the implementation plan that was set up in phase 2 will be executed and rolled out over the 2 subunits according to the schedule in Figure 1. A part of this plan will be the organization of informal small-scale meetings at work wherein the use of the intervention will be demonstrated and explained by the members of the implementation team. In addition, flyers with a brief intervention user manual will be distributed throughout the department.

### Study Procedure

All employees of the participating units will be asked to participate in the intervention evaluation on a voluntary basis. During small-group information sessions, employees will be informed about the study purposes and procedures.

An information letter will show the participants how to install a smartphone-based questionnaire app, after which they will be asked to activate a user account by filling in a unique login username and password. Information letters will be handed out blindly, ensuring that user accounts are anonymous and only used for linking the participant data of different measurement moments (ie, T0, T1, T2, and EMA data). After logging in, the employees will be directed to the baseline questionnaire. The first question of the baseline questionnaire refers to the informed consent and asks the employees if they agree to participate. If they do not agree, the questionnaire will be ended. If they do agree, the participant acknowledges that the information provided was clear. No further follow-up of the nonparticipants will be performed. In addition, during the information sessions and in the information letter, it is indicated that participants are free to quit the study whenever they wish to do so, without having to provide an explanation.

### Intervention Evaluation

#### Web-Based Survey

Intervention effects will be evaluated using pretest (T0/T1) and posttest (T1/T2) web-based surveys (see Figure 1). Participants will be invited to complete the surveys through the smartphone-based questionnaire app. The primary outcome variables used are as follows.

1. Recovery during work: Recovery during work will be measured using 3 items, which reflect the cognitive, emotional, and physical dimension of detachment from work [31,33]. A sample item is as follows: “During a work break, I think of things other than work.” Items will be scored on a 5-point frequency scale ranging from 1 (never or very rarely) to 5 (very often or always).

2. Need for recovery: The need for recovery will be assessed with the “need for recovery scale” from the Dutch questionnaire on the experience and evaluation of work (Dutch abbreviation, VBBA) [34]. A sample item is “Often, after a day’s work, I feel so tired that I cannot get involved in other activities.” All items have 2 response choices: yes or no.

3. Fatigue: Fatigue levels will be measured using the 4-item shortened version of the Checklist Individual Strength [35]. A sample item is as follows: “I feel physically exhausted.” Items will be scored on a 7-point scale ranging from 1 (completely disagree) to 7 (completely agree).

The secondary outcome variables are as follows.

1. General perceived health: The general perceived health will be measured using 1 item of the Dutch version of the short form 36-item Health Survey [36].

2. Vigor: Vigor will be assessed using 3 items of the vigor scale of the Utrecht Work Engagement Scale [37]. A sample item is as follows: “At my work, I am bursting with energy.” All the items will be scored on a 7-point rating scale, ranging from 0 (never) to 6 (every day).

3. Work ability: Work ability will be measured using 3 items of the Work Ability Index [38]. One item is used for subjective estimation of the current work ability compared with lifetime best work ability (11-point scale, ranging from 0 [not able to work at all] to 10 [best work ability in lifetime]), whereas the other 2 items assess the subjective work ability in relation to the physical and mental demands of the work (5-point scale, ranging from 1 [very poor] to 5 [very good]).

4. Safety and performance: Safety and performance at work will be assessed with a self-constructed item for each type of shift (ie, morning/evening/night). A sample item is as follows: “Given the current working conditions, I manage to work safely and productively every morning shift.” The items will be scored on a 7-point scale ranging from 1 (completely disagree) to 7 (completely agree).

The other confounding and effect-modifying parameters that will be collected at baseline are as follows.

1. Sociodemographic variables: Information on age, gender, work unit, job title, and household composition will be collected.

2. Morningness-eveningness preferences: Personal morningness-eveningness preferences will be measured using 1 item based on the Morningness-Eveningness questionnaire [39].

3. Lifestyle: Physical activity and smoking behavior will be measured with 1 item each, based on the Study on Transitions in Employment, Ability, and Motivation cohort study [40].

4. Sleep patterns: Sleep disturbances will be measured using the Jenkins Sleep Questionnaire [41]. This questionnaire consists of the following 4 items: frequency in the difﬁculty of falling asleep in the previous month, difﬁculty sleeping continuously, waking up several times each night, and waking up feeling tired and worn out after the usual amount of sleep. The response alternatives range from 1 (not at all) to 6 (22-31 days).

#### EMA

During the intervention period, all employees will also be asked to participate in the EMA. EMA is a method for self-monitoring through the collection of self-reports on indices of behavior, cognition, or emotions in near real time in the daily lives of the participants, often through digital devices [42-44]. The smartphone-based questionnaire app allows us to digitally collect daily life momentary assessments of changes in employees’ levels of fatigue and recovery behavior, in relation to momentary context and activity. As such, it provides insight into the short-term, within-person mechanisms linking fatigue and recovery with short-term intervention effects. Event-contingent data collection protocols will be used, indicating that data entry takes place when a predefined event occurs [45,46]. The predefined event in this study is the formal work break. Participants will be asked to fill the data in the EMA app for a period of 14 consecutive days (1) directly before taking a work break, (2) directly after taking a work break, and (3) directly after the end of their shift. Each measurement moment will take about 1-2 minutes. In alignment with organizational management, we chose a period of 14 days to ensure enough day-level data points (despite rotating shift schedules and possible dropouts) without overburdening the participants. The use of the intervention, however, can continue after data has been collected.

Table 1 shows the variables that will be measured. The information provided will give insight into the effectiveness of the different recovery strategies (eg, relaxation and activation) and the influence of work break control, shift types, and personal characteristics (eg, age, chronotype, job position) on recovery outcomes. The start of the 14-day EMA period will be communicated through (1) the app via prompts, (2) the manager of the specific unit, and (3) through communication at the workplace (eg, posters, newsletters).

**Table 1:** Variables retrieved through EMA.

Variable	Items	Questionnaire	Before break	After break	After shift
Shift type	1	What shift are you working today?	✓		
Sleepiness	1	Karolinska Sleepiness Scale [47]: scored on a scale from 1 (extremely alert) to 10 (very sleepy, difficulty in staying awake)	✓	✓	✓
Stress	1	Self-developed: “How do you currently feel?” scored on a visual analog scale (ranging from “stressed” to “relaxed”)	✓	✓	✓
Need for work break	1	Self-developed: “To what extent are you in need of a work break right now?” scored on a scale ranging from 1 (not at all) to 10 (to a great extent)	✓		
Detachment from work	3	Based on de Jonge et al [33]: scored on a scale ranging from 1 (completely disagree) to 10 (completely agree)		✓	
Recovery activities	2	Self-developed: “During this work break, I spent my time mainly…”. 1 item scored with 3 answering categories: 1 (on activation activities such as walking, exercising, playing a game); 2 (on relaxation activities such as sitting, reading, listening to music, and power nap); and 3 ((other activities (please specify)) 1 item scored with 2 answering categories: 1 (alone) and 2 (with others).		✓	
Control	1	Self-developed: “I was able to decide for myself how to spend my time during this work break” scored on a scale ranging from 1 (completely disagree) to 10 (completely agree)		✓	
Work break satisfaction	1	Self-developed: “I am satisfied with the way I spend my time during this work break” scored on a scale ranging from 1 (completely disagree) to 10 (completely agree)		✓	
Level of recovery	1	Based on Demerouti et al [20]: “At this moment, I feel sufficiently recovered” scored on a scale ranging from 1 (completely disagree) to 10 (completely agree)		✓	
Productivity	1	Self-developed: “To what extent were you able to work productively during this shift?” scored on a scale ranging from 1 (not at all) to 10 (a great extent)			✓
Safety	1	Self-developed: “To what extent were you able to work safely during this shift?” scored on a scale ranging from 1 (not at all) to 10 (a great extent)			✓

#### Process Evaluation

It is not only important to develop an intervention with the right ingredients but also to design a good implementation process [48]. Studies have shown that the intervention development and implementation process can affect the outcomes of the respective intervention [27,28]. For instance, activities aimed at raising support from the employees and the management for the intervention may enhance the effectiveness of an intervention. However, failing to fully implement a planned intervention after raising support may result in unfulfilled expectations and negative employee attitudes. By evaluating the intervention process, the outcomes of organizational interventions can be better understood [48,49]. These insights can be used to further improve the intervention effectiveness.

In this study, we will use Nielsen and Randall’s [50] framework for process evaluations, which is specifically designed for the implementation process of organization-level interventions. It provides a broad perspective on the intervention process by not only including the intervention design and implementation but also the organizational context and the participant’s perceptions of the intervention. Evaluation of these factors will enable us to answer the questions of what works for whom under which circumstances [48]. Data on the process factors will be collected during and after the implementation of the intervention. A variety of data sources will be used: (1) data logs of the researchers and the organizational management, (2) interviews with the management and the employees, and (3) the T1/T2 web-based employee survey. Table 2 provides an overview of the process factors, associated questions, and data sources.

**Table 2:** Overview of the process evaluation factors.

Process factor		Question	Data logs	Interviews	T1/T2^a^ survey
**Intervention design and implementation**					
	Initiation	Who initiated the intervention and for what purpose?	✓	✓	
	Developing intervention activities	Did the intervention activities target the problems of the workplace?	✓	✓	✓
	Implementing intervention activities	Did the intervention reach the target group?	✓	✓	✓
**Implementation strategy**					
	Drivers of change and the roles of key stakeholders	Who were/are the drivers of change?	✓	✓	
	Employee involvement	Did employees participate significantly in decision making and how many were involved?	✓	✓	✓
	Management support	What was the role of the senior/middle managers?	✓	✓	✓
	External consultants	What was the role of the external consultants? *(Not Applicable)*			
	Information and communication	What kind of information was provided to the participants during the study?	✓	✓	✓
**Context**					
	Omnibus context	How did the intervention ﬁt in with the culture and the conditions of the intervention group?	✓	✓	✓
	Discrete context	Which events took place during the intervention phase?	✓	✓	
**Mental models**					
	Readiness for change	To what extent are/were the participants ready for change?		✓	✓
	Shared mental models	To what degree do the participants have shared mental models?		✓	✓
	Appraisal of the intervention and its activities	How did the participants perceive the intervention and its activities?		✓	✓
	Changes in mental models	Did the intervention bring about a change in the participants’ mental models?		✓	✓

^a^T1/T2: Posttest web-based survey.

### Statistical Analysis

First, intervention results will be analyzed by performing mixed model repeated measures analyses (multivariate analysis of variance; time*group interaction) on longitudinal survey data with the outcome measures at follow-up (T0-T1, T1-T2, and T0-T2 comparisons) as the dependent variables. Furthermore, the effectiveness of the intervention will be analyzed by applying multilevel analyses on data collected within the EMA study (before break-after break and before break-after shift comparisons). Data will be collected and analyzed at 3 levels: (1) day level, (2) employee level, and (3) work unit level. Dropouts will be documented and included in the data analysis to the point of dropout. Potential confounders or effect modifiers (eg, age, gender, chronotype) will be compared between the intervention and the waiting list control group by *t* tests for independent samples and chi-square tests. For all analyses, a two-tailed significance level of *P*<.05 will be applied. The multilevel analyses will be conducted using R and the mixed model repeated measures analyses will be performed using SPSS software (IBM Corp, Version 25.0). A detailed analysis plan will be developed prior to finalization of the dataset.

### Sample Size

The sample size calculation is based on finding an effect on the need for recovery. This variable was chosen because of its test-retest reliability and sensitivity to detect change, indicating that the need for recovery may be a useful tool for evaluating interventions related to occupational health [34]. Based on previous studies [47,51], a small effect size is expected. Power calculations indicate that to detect a small effect in the context of a repeated measurements analysis of variance (Cohen *f*=0.15), at least 37 subjects are necessary in each study group (power=0.80 and α=.05), and calculations were performed using G*power [52]. The response rates of similar PAR studies have been shown to be around 75% or higher [31,32]. Given the intended sample size of 150 subjects and the expected response rate of 75%, resulting in 56 subjects per group, the study has sufficient power to even detect slightly smaller effects [53].

## Results

This study is supported by the Netherlands Organization for Applied Scientific Research Work and Health Research Program, which is funded by the Ministry of Economic Affairs and supported by the Dutch Ministry of Social Affairs and Employment, program number 19.204.1-3. This study was approved by the institutional review board on February 7, 2019. From June to August 2019, baseline data were collected, and from November to December 2019, the first follow-up data were collected. The second follow-up data collection and data analysis are planned for the first two quarters of 2020. Dissemination of the results is planned for the last two quarters of 2020.

## Discussion

### Principal Aspects of This Study

Shift work (ie, irregular working hours) can cause negative short-term effects for both employees and employers, such as increased fatigue levels, concentration problems, and consequently, an augmented risk of accidents and productivity loss. Unfortunately, evidence-based interventions to mitigate the negative effects of shift work are still lacking. Previous research studies have shown that a possible way to counteract the negative short-term effects of shift work is by enhancing the recovery of the employees during work. Therefore, the aim of this study is to develop, implement, and evaluate an intervention focused on enhancing the recovery of shift workers during working hours.

### Study Strengths

The design of this study has several strengths. First, by making use of the PAR approach, stakeholders at all levels of the company are involved. This bottom-up involvement of the stakeholders will contribute to the commitment to the proposed interventions, which will likely translate into better overall intervention adherence and compliance. Moreover, it allows the target group to participate in the development and implementation of the intervention. This approach will stimulate problem ownership and commitment at all levels of the organization and has the potential to contribute to organizational sustainability [29], for which the interventions are more likely to have the proposed impact [28]. Another strength of the study design is that the timeline for the experimental unit allows us to compare the short-term intervention effects (T1) with the long-term effects (T2). In addition, the intervention development is conducted in a similar way in both the departments, but the eventual workplace interventions can differ. As a consequence, we can compare different solutions on similar proposed outcomes, further contributing to both theory and evidence-based practice [54]. In addition, to determine the effects of the workplace intervention, we use a rather unique data triangulation. Next to group-level outcome measurements with a web-based questionnaire, EMA measurements are used to gain insight into the working mechanisms of the intervention and to determine the influence of the type of recovery strategy chosen, the time of day, the type of shift, and intraindividual differences. Moreover, a process evaluation will be performed in order to gain more insight into what aspects of the intervention work for whom and why [48]. The process evaluation outcomes are assessed qualitatively (ie, group interviews, observations, data logs) and quantitatively (ie, self-reported measures in digital surveys) and whenever possible, complemented with objective organizational data (eg, sickness absence registration).

### Study Limitations

From an occupational and epidemiological point of view, this study can be classified as a prevention-effectiveness study [27,55,56]. The characteristics of a prevention-effectiveness study design are as follows: small sample size, no randomization or blinding, and quantitative and qualitative measures. These characteristics ensure the internal validity of the study [55]. However, this type of study, as does ours, contains some limitations as well. First, because we do not use randomization, allocation bias could take place. However, in this study, it is impossible to randomly assign participants to an intervention and control group for practical and ethical reasons. Therefore, the waiting list control group principle was introduced. Moreover, one could argue that owing to the preallocation of the departments, the commitment of the management to the study is assured (ie, they know that an actual intervention is taking place and things have to be arranged to get this done), which enhances the feasibility of this study. A second limitation is the timeframe of the study. Behavioral and organizational changes do not occur easily or quickly. Therefore, the follow-up period (6 months) might be too soon to establish health-related effects. It might be, for instance, that changing recovery habits initially requires additional effort before it pays off in long-term reduced levels of fatigue. By combining quantitative data with qualitative data, we aim to gain further insight into this matter. A third limitation is that the evaluation of the effectiveness of the intervention fully relies on self-reported data. It would be interesting, for instance, to also assess fatigue levels through simple cognitive tests. For reasons of feasibility (ie, extensiveness of current research activities and accompanying time investment from the participants), we did not include such tests in the current design, but it might be a useful addition to the EMA method in the future studies. Another study limitation is the lack of generalizability of the findings. These study results may be organization-specific or sector-specific. However, workplace intervention studies for shift workers are still very scarce. The intervention in this study may therefore offer a starting point for future intervention studies in other organizations and sectors with shift work. Thereafter, aspects of the intervention should again be tailored to the workplace-specific context and target group through a participatory development and implementation process, as this has been argued to be a crucial condition for organizational interventions to be effective. An inevitable implication of this approach is that the effects have to be interpreted by considering unique contextual factors. A process evaluation will be performed to identify such contextual factors.

In summary, this study will investigate whether a workplace intervention study can reduce fatigue and improve the recovery of shift workers during work by using a PAR-designed workplace intervention at a large steel company in the Netherlands.

## Abbreviations

EMA: ecological momentary assessment
HR: human resource
PAR: participatory action research
